# IR microspectroscopic investigation of the interaction of some losartan salts with human stratum corneum protein and its effect on losartan transdermal permeation

**DOI:** 10.1371/journal.pone.0287267

**Published:** 2023-06-15

**Authors:** Randa S. H. Mansour, Aamal Y. Al Khawaja, Imad I. Hamdan, Enam A. Khalil

**Affiliations:** 1 Faculty of pharmacy, Philadelphia University, Amman, Jordan; 2 School of pharmacy, University of Jordan, Amman, Jordan; Siksha O Anusandhan University School of Pharmaceutical Sciences, INDIA

## Abstract

The interaction of pharmacologically active drugs with SC biochemical components is underestimated in pharmaceutical research. The aim of this research was to illustrate that some drugs intended for transdermal delivery could interact with the protein component of SC. Such interactions could be in favor of or opposition to their percutaneous absorption. IR microspectroscopy was used to delineate possible interaction of SC keratin with three losartan salts LOS-K, LOS-DEA and LOS-AML salts in addition to AML-BES salt. The results of PCA, combined with comparisons of average second derivative spectra of SC samples treated with these salts and the control SC, showed that LOS-DEA did not interact with SC, thus providing base line permeation of losartan. AML-BES, LOS-AML and LOS-K salts modified the conformational structure of keratin. The disorganization effect on the α-helical structure and induced formation of parallel β-sheets and random coils were in the order of AML-BES˃LOS-AML˃LOS-K. The order of the impact of treatments which resulted in increased formation of β-turns was AML-BES˃LOS-AML. The formation of antiparallel β-sheets was manifested by LOS-AML. Thus, the overall effect of these salts on the SC protein was AML-BES˃LOS-AML˃LOS-K. The impact of LOS-K was associated with improved permeation whereas the impact of LOS-AML was associated with hindered permeation of both losartan and amlodipine. There is a possibility that losartan and amlodipine when present in combination inside SC, their binding to the protein is enhanced leading to being retained within SC.

## 1 Introduction

The layered structure of the skin is well identified with the outermost 10–20 μm thick layer being the stratum corneum (SC) [1]. Having a unique structure and organization, the SC presents the primary barrier against the absorption of substances through the skin. According to the classic brick and mortar model, SC is composed of keratin-rich corneocytes, considered the bricks, embedded in a lipid intercellular matrix (ceramides, cholesterol and free fatty acids) organized as lamellar lipid layers and considered the mortar [2, 3].

Some chemical substances are capable to interact with intercellular lipid or intracellular protein components of SC. Taking into consideration the published research in this context, three categories of those substances can be recognized: chemical penetration enhancers, topically applied cosmetic and personal care products, and active ingredients in medicinal products.

Chemical penetration enhancers are the most commonly studied ones as they are intended to overcome the barrier properties of SC in order to enhance the percutaneous penetration of drugs thus, facilitating transdermal delivery of therapeutic agents. Many studies utilizes infrared (IR) microspectroscopy to identify the effect of chemical penetration enhancers, such as oleic acid [4], other fatty acids [5] and Emu oil [6] on the lipid and/or protein constituents of SC. Furthermore, other techniques such as synchrotron X-Ray diffraction [7] and neutron diffraction [8] were used to investigate the interaction of penetration enhancers with SC lipids. The effect of some nontraditional penetration enhancers on the biochemical composition of SC was also investigated. One example is a unique vaccine delivery system through skin, modified immune-stimulating complexes (ISCOMs). ISCOMs were found to interact with a SC lipid model similar to the intercellular lipid domain of SC. Förster resonance energy transfer and atomic force microscopy, among other techniques [9] were used to study the interaction. The interaction of different phospholipids systems with SC lipids has been also addressed to reveal their permeation enhancing effects using IR spectroscopy [10] or other approaches such as fluorescence anisotropy [11].

To a lesser extent, the interaction of topically applied cosmetic products, particularly moisturizers, with SC components is also present in literature. Such interaction may lead to compromised barrier properties of SC or increased susceptibility of skin to irritation, which could be attributed to specific components of the moisturizers [12]. The interaction of personal care product ingredient, sodium dodecyl sulphate, with human SC was also investigated by IR spectroscopy [13]. In another study, the objective was to investigate the interaction of antiseptic quaternary ammonium compounds with rat SC lipid components in an attempt to clarify the mechanisms of their permeation into SC. Some of the investigated compounds were able to bind to cholesterol and thus accumulate in SC, others were able to bind to and subsequently extract cholesterol from SC whereas some compounds were concluded to interact with ceramides [14].

Very limited amount of research was devoted to investigate the interaction of pharmacologically active drugs in medicinal products with SC lipids or proteins. Indeed, one can barely find examples. In one study, miltefosine, a drug used to treat visceral leishmaniasis, was found to interact with SC lipid component leading to an increase in its fluidity, an effect that could be utilized for the development of topical treatment of cutaneous leishmaniasis. The study was performed on SC of rats by electron paramagnetic resonance spectroscopy and fluorescence spectroscopy [15]. In a recent study, an antifungal drug, terbinafine, was found to interact with SC protein component. The interaction was assessed by IR spectroscopy showing the ability of the drug to induce substantial β-sheet formation [16]. Similarly, hyaluronic acid interaction with protein and lipid components of pig SC was studied using ATR-IR spectroscopy [17].

On the other hand, we have recently investigated the potential transdermal delivery of a novel salt of an antihypertensive drug losartan, losartan amlodipine (LOS-AML), as an alternative for the conventional available oral delivery of the potassium salt (LOS-K) [18]. LOS-AML salt was thought to be a good candidate for transdermal permeation compared to LOS-K since it has favorable physicochemical properties. LOS-K is considered to have higher hydrophilicity than that required to achieve satisfactory percutaneous permeation of losartan, whereas LOS-AML has higher lipophilicity, lower water solubility, lower melting temperature and higher partition coefficient. At the same time, amlodipine, the positive counterion in LOS-AML, acts also as a hypertensive agent, hence the choice of this specific salt. Thus LOS-AML constituted a “drug-drug salt” that was thought to offer pharmaceutical benefits as the topic is increasingly gaining more interest [19, 20]. However, the performed diffusion studies through human SC, from an aqueous solution based on propylene glycol (PG) revealed vast decrease of permeation of LOS from the molecular salt form LOS-AML in comparison to LOS-K. Similarly, a decrease in permeation was also noted for amlodipine as compared to that of amlodipine besylate (AML-BES) form. The study concluded that the salt structure was maintained during permeation and potential drastic retention of the salt molecules in SC was suggested based on the results of the diffusion studies [18]. Possibly, strong chemical interaction between the salt and the constituents of SC could have lead to this effect.

The scarcity of studies dealing with the interaction of drugs with SC components, and the fact that the limited available research within this context is focused on topically applied drugs, combined with the results obtained from investigating the percutaneous permeation of LOS-AML, have urged us to investigate the interaction of SC with LOS-AML. The occurrence of such interactions is critical since it may have an impact on the drugs retention capability within the SC or their diffusion behavior through it.

The investigated drugs were LOS-K, AML-BES, in addition to our novel salts, LOS-AML and losartan diethyl amine (LOS-DEA). The effect of LOS-AML on the protein structure of SC was compared to that of LOS-K and AML-BES. Furthermore, the effect of LOS-DEA was also investigated to reveal whether other losartan salts have the ability to interact with SC or not. IR microspectroscopy combined with multivariate statistical analysis was used to delineate the effect of the applied salts on the protein components of human SC.

## 2 Methods

### 2.1 Materials and equipment

LOS-K and AML-BES were kindly donated by Jordan Pharmaceutical Manufacturing (JPM, Amman, Jordan) and Al Hayat Pharmaceutical Industries Company (HPIC, Amman, Jordan) respectively. The investigated novel salts prepared in our laboratory were LOS-AML [18] and losartan diethyl amine (LOS-DEA). LOS-DEA was prepared by reacting LOS-K with an equimolar amount of diethylamine using the following procedure: 3g of LOS-K were added to 10 ml of distilled water and heated at 50°C until complete dissolution. The required volume of DEA (0.67 mL) were dropwise added to the resulting solution. The salt was then obtained by salting out using saturated solution of sodium chloride, filtered using gravity filtration process and placed into a desiccator filled with fresh calcium chloride granules for drying. The dried salt was grinded and filled in stored tightly closed for not more than one weak for further characterization. (Refer to Supplementary data for details of characterization).

PG was obtained from SIGMA-AlDRICH^®^, Darmstadt, Germany. Trypsin was obtained from SIGMA-ALDRICH, USA.

ThermoNicolet 8700 spectrometer coupled with a continuum infrared microscope at IR laboratory of SESAME synchrotron (Jordan) using the globar source was used for IR data measurement. The microscope is equipped with a liquid nitrogen-cooled mercury cadmium telluride detector and OMNIC 9.1 software (Thermo Fisher scientific) for data acquisition for the SC samples. The CaF_2_ windows were obtained from Crystran Ltd, UK whereas the diamond compression cell was obtained from Specac, UK.

### 2.2 Preparation of the treatment solutions

Sixty percent PG in phosphate buffer saline (pH 6.8) was used as a solvent to prepare the treatment solutions of the investigated salts. Solution of each investigated salt of losartan (LOS-K, LOS-DEA and LOS-AML) was prepared at a concentration equivalent to 80% of losartan solubility in each corresponding salt [17, S1 File]. Similarly, a solution having a concentration equivalent to 80% of amlodipine solubility in AML-BES salt was prepared [18]. A treatment solution of 60% PG was also prepared to provide a control treatment.

### 2.3 Preparation of human skin sheets and isolation of SC

The study was approved by the Ethics Committee for Scientific Research on Humans at Philadelphia University. Human skin of a 44-year-old female donor undergoing abdominal plastic surgery was obtained from a local hospital immediately after the surgery. The research objective was explained to the donor and her approval was verbally granted directly, since she owned the samples after conducting the surgery and had the option to bury them or donate to scientific research. Her approval was granted at two different occasions, before and after conducting the surgery in the presence of her surgeon. The obtained skin was appropriately processed and stored. Three circular discs of 25 mm in diameter for In-vitro permeation experiment in addition to six circular discs of 12 mm in diameter for IR microspectroscopic analysis were punched out from the frozen human skin sheet. The discs were left for few minutes to thaw then soaked in 1% w/v trypsin in distilled water at 32°C for 24 hr to isolate the SC. The isolated SC was soaked for 2 hr in distilled water to wash.

### 2.4 In-vitro permeation of LOS-DEA

In-vitro permeation of LOS-DEA was performed as described in reference 18. The total cumulative permeated amount of LOS after 24 hr of permeation (Q_24_) in μg was obtained from the permeation plots and the results were reported as average ±SD. The obtained values were statistically compared to those of LOS-K and LOS-AML [17] using one-way analysis of variance (ANOVA) at 0.05 level of significance, followed by Bonferoni multiple-comparison test employing SPSS Statistics 17.0 software.

### 2.5 FTIR isolated SC sample preparation

The upper surface of four SC pieces was swabbed three times with a cotton swab impregnated with the corresponding treatment solution (LOS-K, LOS-DEA, LOS-AML or AML-BES in 60% PG) (section 2.2). The fifth piece was swabbed by the same manner with 60% PG to serve as a control. All the solutions were previously equilibrated at 32°C. The sixth piece was left untreated. The SC pieces were then incubated at 32°C for 24 hr. To remove the residual treatment solutions, the pieces were then gently pressed between filter papers and the surfaces were washed by swabbing with a cotton swab impregnated with distilled water and gently pressed between filter papers. The SC pieces were then mounted on CaF_2_ windows and kept for 30 min in a closed chamber purged with compressed dry air to accomplish drying. Analysis of the SC samples by the IR microscope was immediately started thereafter.

### 2.6 FTIR microspectroscopy

All IR measurements were carried out at SESAME synchrotron (Jordan). IR microscopic map for each isolated SC sample was acquired using the 15X Schwarzschild objective of the IR microscope. All spectra were obtained using a double path single masking aperture size of 40* 40 μm^2^ at step size of 20 μm, and a spectral resolution of 2 cm^-1^. For each spectrum, 256 co-scans were recorded in the mid-infrared range between 4000 and 700 cm^−1^ in transmission mode.

In addition, a representative spectrum for each of: LOS-K, LOS-DEA, LOS-AML, AML-BES and PG was obtained after being deposited into diamond compression cell using the same described settings.

### 2.7 FTIR data treatment and statistical analysis

Splitting of the obtained IR maps was done by Omnic software to yield a data set corresponding to each SC sample. The resultant raw spectra (100–150) of all data sets were preprocessed in the spectral domain corresponding to protein amide I and II vibrations using the Unscrambler 10.3 software (CAMO software). In this domain, the spectra were appropriately smoothed and base line corrected. Savitsky-Golay second derivatives were then calculated using third polynomial order and seven smoothening points, and subsequently range normalized. Principal component analysis (PCA) was performed and the results were represented in the form of score and loadings plots. The unit vector normalized reduced average spectra and their corresponding range normalized Savitsky-Golay second derivative (third polynomial order and seven smoothening points) of the data sets were obtained to compare the effect of the different treatments in accordance with the PCA results. Furthermore, the average Savitsky-Golay second derivative of each data set was range normalized with that of its corresponding treatment (LOS-K, LOS-DEA, LOS-AML, AML-BES or PG) and carefully compared.

## 3 Results and discussion

Amide I and Amide II bands constitute the main IR spectral features of SC that correspond to its protein content. Amide I feature arises mainly from C = O stretching of amide groups of the peptide backbone in proteins and is located at around 1650 cm^-1^, whereas amide II feature arises mainly from N–H bending and C-N stretching vibrations of amide groups of the peptide backbone in proteins and is located at around 1550 cm^-1^. Both bands, particularly the amide I band, are sensitive to protein secondary structure [21–25], thus are used to delineate the alterations in the secondary structure of SC protein.

Keratin is the main protein constituent of human SC, which is predominantly present in α-helix secondary structure and is known to significantly contribute to the Amide I band. Ceramides and other proteins provide minor contribution to this band, consequently, it is indicative to keratin structural changes [26, 27].

**Fig 1:**
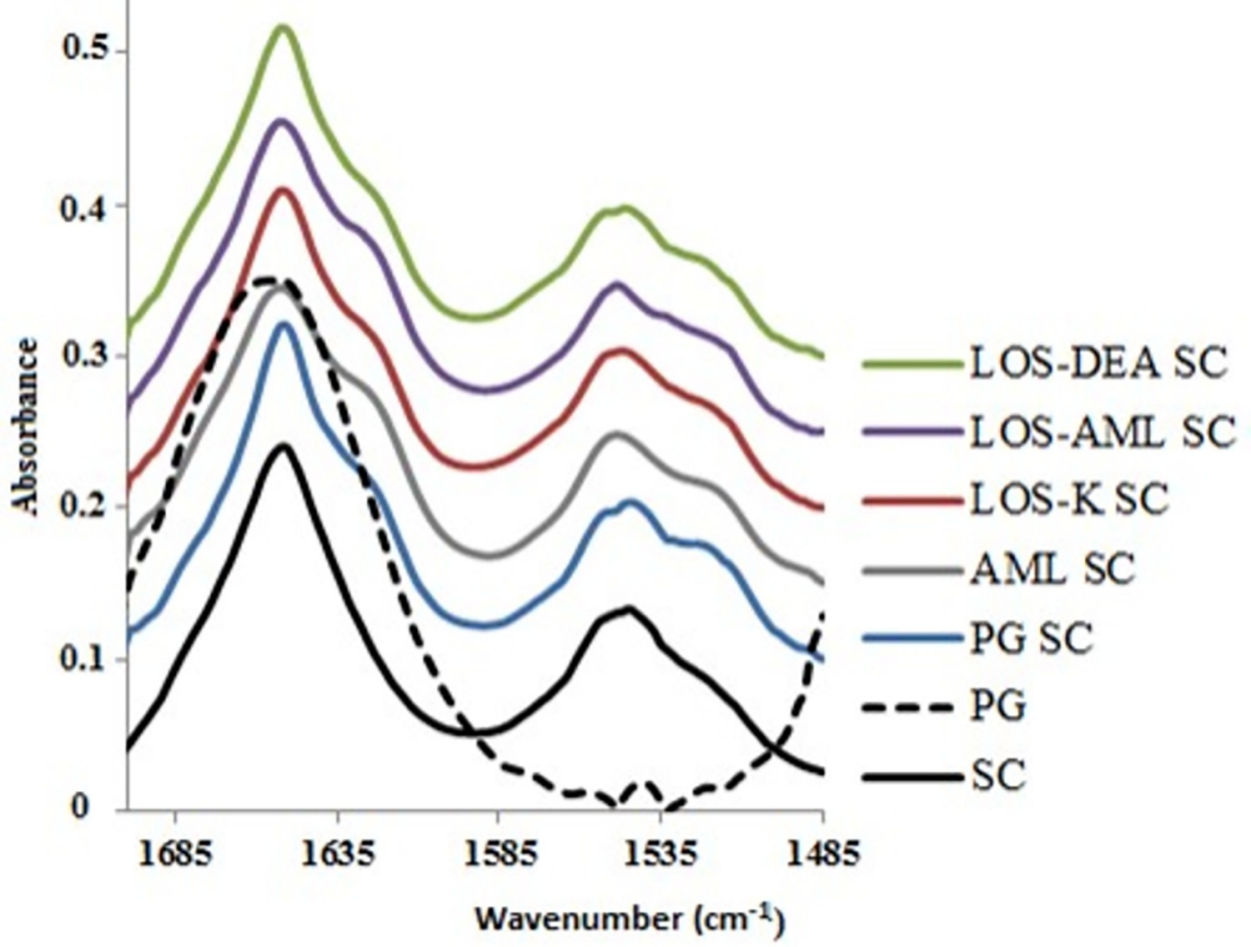
Unit vector normalized average spectra of all treated isolated SC samples, untreated SC and neat PG, showing protein amide I and II peaks (The spectra are offset for clarity).

Fig 1 illustrates the unit vector normalized average spectra of PG, LOS-K, LOS-DEA, LOS-AML, AML-BES treated isolated SC samples, designated as PG SC, LOS-K SC, LOS-DEA SC, LOS-AML SC and AML-BES SC respectively. The protein amide I and II peaks are clearly shown. The treated SC samples exhibited the amide I peak at 1650.8 cm^-1^ except for AML-BES treated sample in which a shift to higher frequency (1652.7 cm^-1^) occurred. A shift of the amide I band to a slightly higher frequency is consistent with a decrease in hydrogen bond strength of the peptide bond [6].The obtained results confirm the dominant helical structure of keratin. Furthermore, variation in the peak contour between the samples can be noted, suggesting possible variation in protein structure. On the other hand, the position of amide II peak is variable ranging from 1542.8 to 1548.6 cm^-1^. In addition, emergence of a shoulder at lower frequency is evident for PG SC and LOS-DEA SC. Fig 1 shows a comparison between PG SC, untreated SC and neat PG unit vector normalized average spectra. Taking into consideration the IR features of neat PG within this spectral range, it is obvious that the differences in the amide II contour of PG SC; as compared to that of untreated SC; can be easily attributed to arise from PG. Interestingly, among the other treated samples, the signs pertinent to the presence of PG were evident only in LOS-DEA SC and were less evident in the others. The shadowing of these signs, despite the use of PG in all treated samples, could be as a result of the other treatments own IR features or features arising from the interaction of the corresponding treatment with SC proteins.

**Fig 2:**
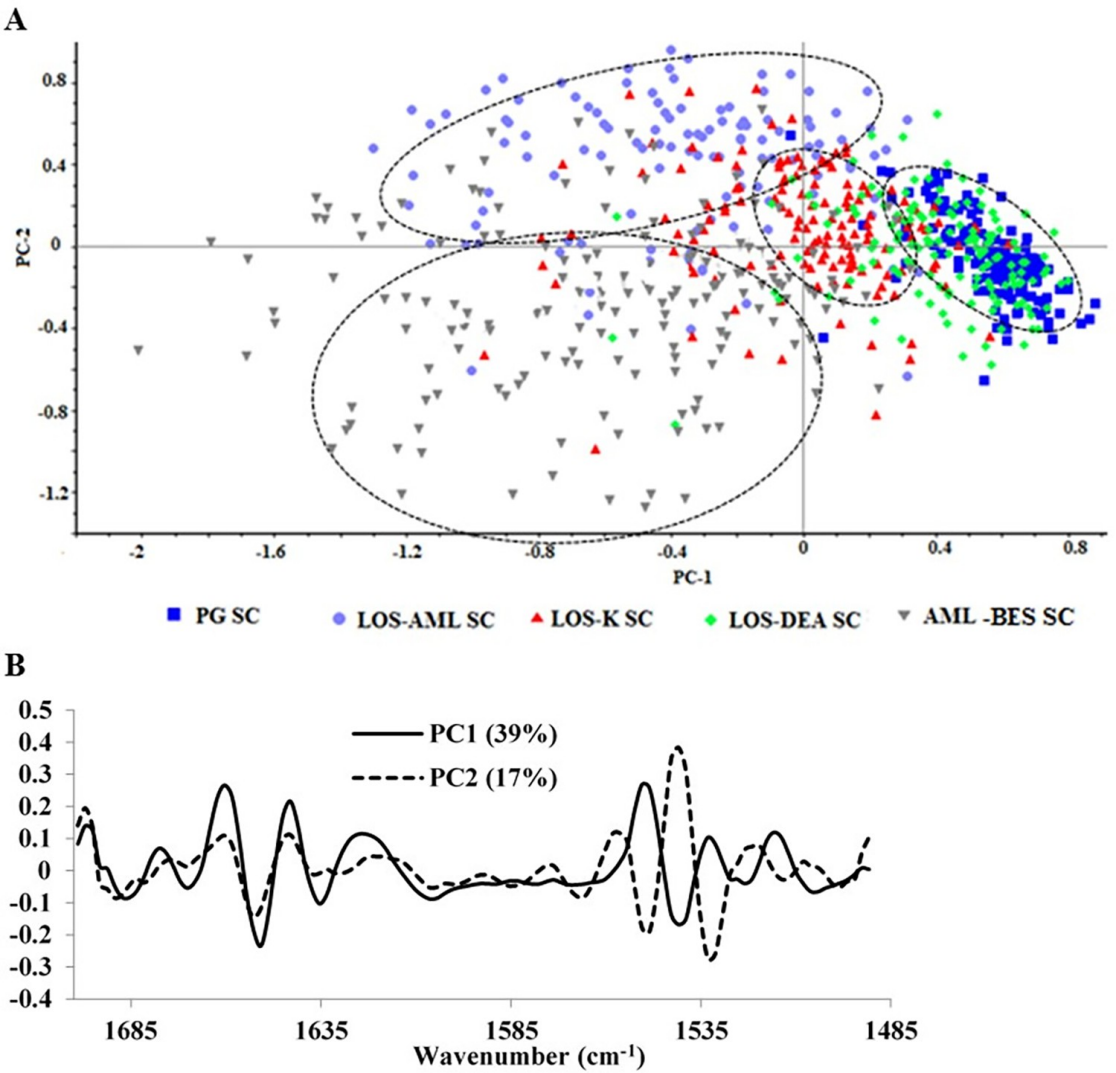
A) Score plot and B) corresponding PC1 and PC2 loadings of protein region.

**Fig 3:**
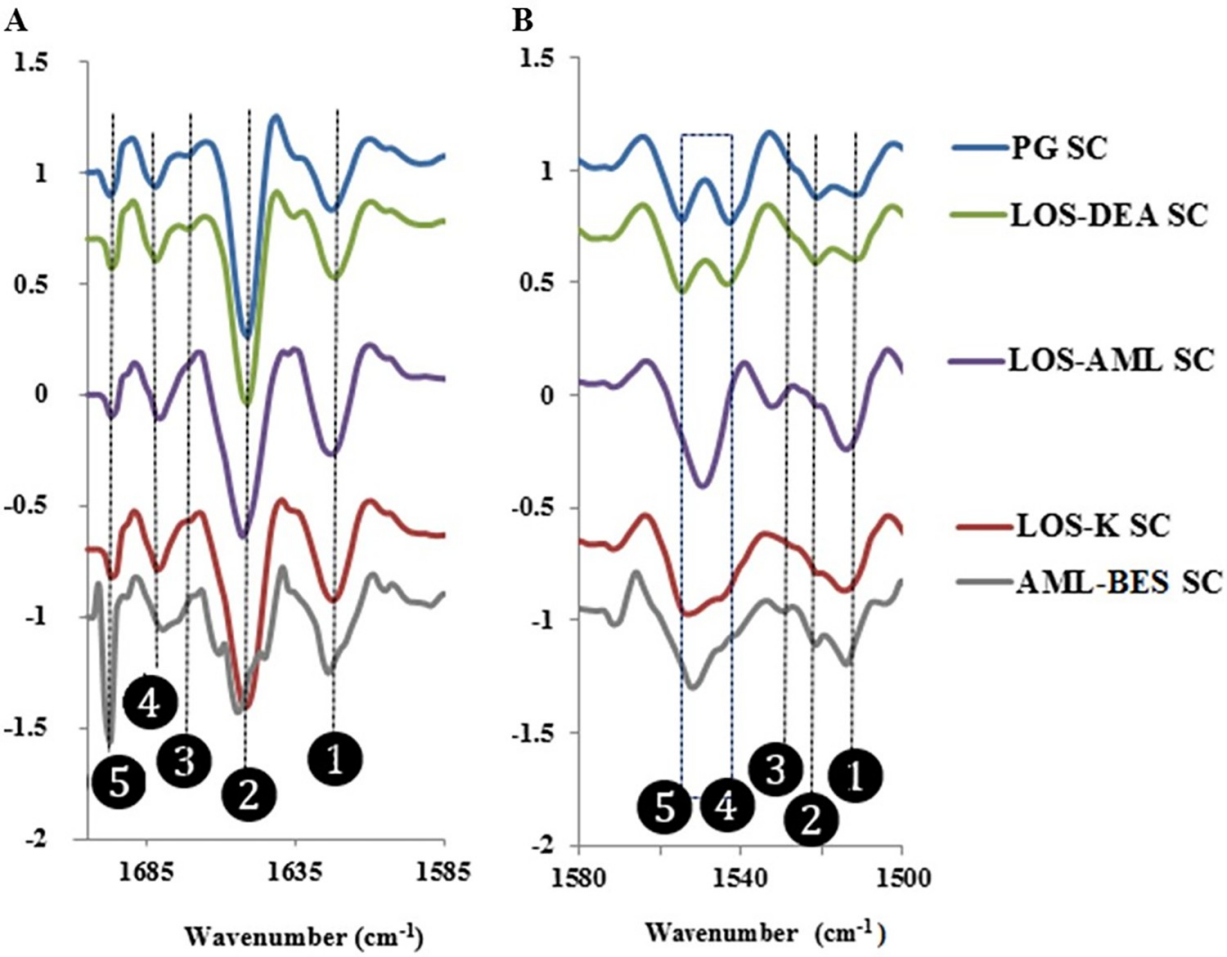
Range normalized average second derivative spectra of A) amide I and B) amide II peaks of the treated SC samples. The spectra are offset for clarity.

The above discussion highlights possible alterations in the secondary structure of keratin, however, it is difficult to manifest and analyze these alterations by analysis of the raw spectra. To identify the overlapping IR bands in the protein spectral region, that correspond to different protein secondary structures, second derivative spectrum analysis provides one of the most popular approaches [23, 28]. Accordingly, PCA was performed on second derivative spectra and the results in form of score and PC1/PC2 loading plots are shown in Fig 2A and 2B respectively. The average second derivative spectra of all samples are depicted in Fig 3A and 3B for amide I and II regions respectively. The trends of clustering or separation in the score plot provided an insight on the intra/inter-sample spectral differences and similarities, whereas the loading plot identified spectral features responsible for intra- and inter-sample variation. The average second derivative spectra were used to compare and explain spectral differences among the samples.

**Fig 4:**
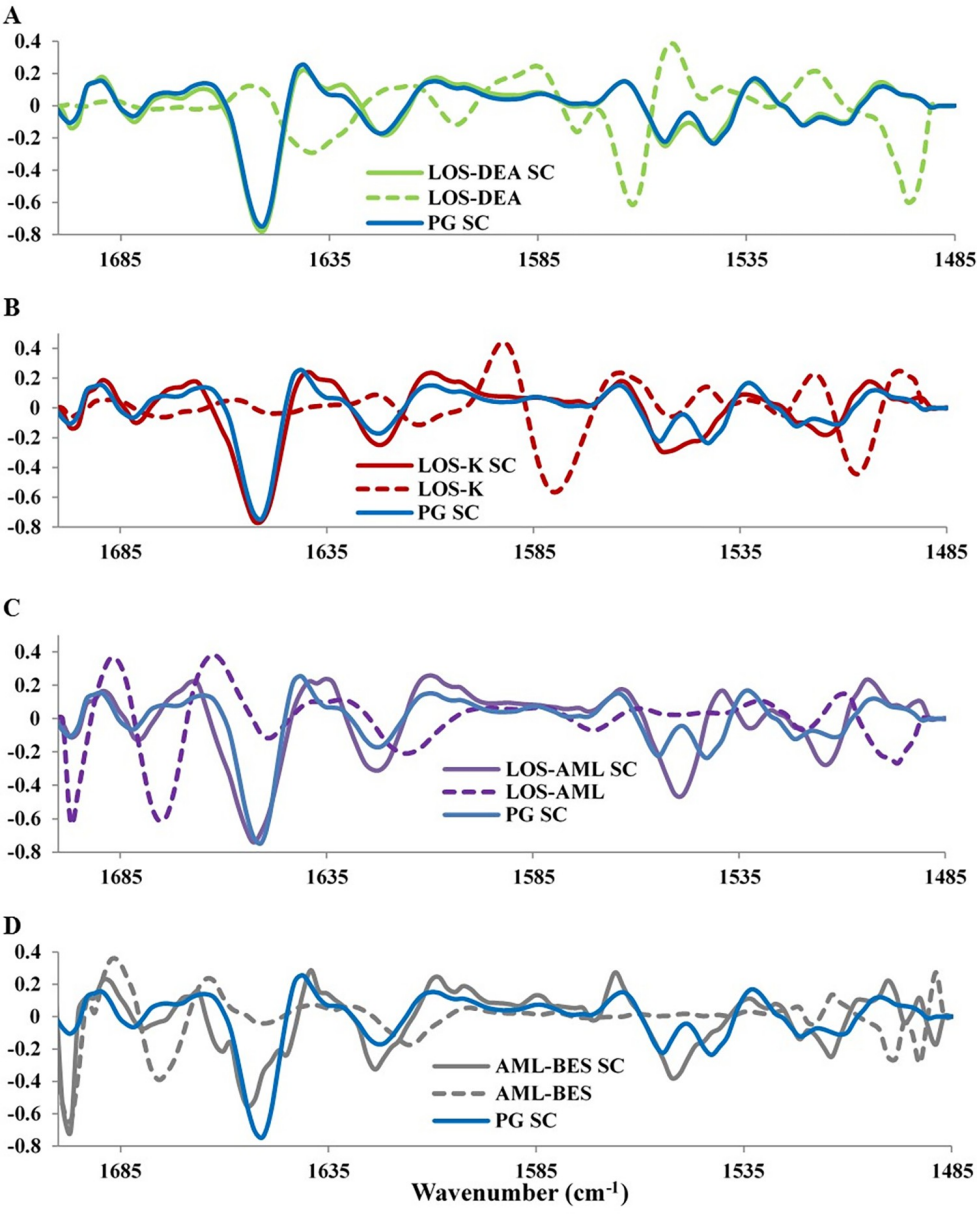
Range normalized average second derivative spectra of amide I and amide II peaks of each treated SC sample in comparison to that of the corresponding exogenous treatment salt and that of PG treated SC sample.

PG SC and not SC was taken as the control sample in order to exclude the spectral differences arising from the presence of PG since PG exhibits IR features within the investigated spectral range (Fig 1B). This procedure is of outmost importance to avoid false interpretation of these PG exogenous features as being caused by modification of protein structure resulting from the corresponding examined treatment. It is also crucial to emphasize that the IR features corresponding to the applied salt treatment could be similarly misinterpreted as variation in protein structure arising from the interaction of the corresponding treatment with SC. To avoid this, the identified peak differences from the average second derivative spectra were then examined in comparison to the exogenous IR signals of each corresponding salt treatment (Fig 4A–4D).

Concerning the score plot (Fig 2A), it is clearly shown that the spectra of LOS-DEA SC lie within the vicinity of those of PG SC; thus reflecting the spectral similarity between those two samples. Each of LOS-K, LOS-AML and AML-BES SC spectral groups is well clustered and separated from those of other samples, hence reflecting inter-sample spectral differences. It can be also concluded that LOS-AML and AML-BES provide more differences from the control PG SC compared to LOS-K as, unlike the other samples, they lie on the negative side of PC1. Moreover, LOS-AML and AML-BES are spectrally very different since they lie on opposite sides of PC2. In addition, the intra-sample variability in LOS-AML and AML-BES SC is higher than that of the others.

The loadings of PC1 and PC2 (Fig 2B) shows that various peaks account for the noticed variability. Those peaks were compared between the samples in amide I and II regions by referring to the range normalized average second derivative spectra (Fig 3). Ten peak positions in amide I and II spectral ranges were found relevant. The inter-sample differences in these peaks are summarized in Table 1.

**Table 1:** Changes in spectral features in the second derivative spectra of SC as a result of the investigated salts.

Spectral Region	Peak No.	Treatment of SC				
		Control (PG)	LOS-DEA	LOS-K	LOS-AML	AML-BES
**Amide I**	1	1621.9 cm^-1^	No change	Higher intensity	Higher intensity	• Shifted to 1623.8 cm^-1^ • shoulder at 1618 cm^-1^
	2	1650.8 cm^-1^	No change	• Peak broadening (slight)	• Shifted to 1652.8 cm^-1^ • Peak broadening (intermediate)	• Shifted to 1654.7 cm^-1^ with high decrease in intensity • Emergence of two shoulder at 1645 and 1660 cm^-1^
	3	1670.1 cm^-1^	No change	No change	Small increase in intensity	Small increase in intensity Shifted to 1673.9 cm^-1^
	4	1681.6 cm^-1^	No change	No change	Shifted to1679.7 cm^-1^	Shifted to 1679.7 cm^-1^
	5	1697.1 cm^-1^	No change	No change	No change	Increased intensity (high)
**Amide II**	1	1510 cm^-1^	No change	• Shifted to 1513.9 cm^-1^ • Increased intensity (slight)	• Shifted to 1513.9 cm^-1^ • Increased intensity (high)	• Shifted to 1513.9 cm^-1^ • Increased intensity (intermediate)
	2	1521.6 cm^-1^	No change	No change	Slight decrease in intensity	No change
	3	1527.4 cm^-1^ (faint shoulder)	No change	No change	• Shifted to 1531.2 cm^-1^ • Increased intensity (high)	• Shifted to 1529.3 cm^-1^ • Increased intensity (slight)
	4 & 5	1542.8 cm^-1^ and 1554.4 cm^-1^ of same intensity	No change	• Shifted to 1544.7 cm^-1^ and • 1552.4 cm^-1^ • The second peak is of higher intensity	One central intense peak at 1548.6 cm^-1^	• Shifted to 1544.7 cm^-1^ and 1552.4 cm^-1^ • The second peak is of higher intensity

In agreement with the score plot, PG and LOS-DEA treated SC samples have noticeable high degree of similarity in the amide I and II regions. On the other hand, Fig 4A shows that there is no overlap between the features of PG SC and those of the applied salt, which could otherwise shadow spectral changes in LOS-DEA SC. These results reflect the inability of LOS-DEA to interact with SC and modify its protein structure.

Within amide I range, Peak 1 is attributed to parallel β-sheets [21–28]. LOS-K and LOS-AML had similar impact on increased formation of this structure as they intensified this peak in comparison to control; nevertheless, the extent of impact is higher for LOS-AML. AML-BES exhibited a different effect on this structure as evident by peak shifting to higher frequency and emergence of new shoulder at a lower frequency at 1618 cm^-1^ usually arising from intermolecular parallel β-sheets.

Peak 2 occurs in the range of the α-helical structure of protein [21–28]. LOS-K caused peak broadening and LOS-AML caused peak broadening associated with peak shifting to a higher frequency in comparison to the control. Some degree of disorganization of this structure is evident by these changes and, similar to the effect on peak 1, LOS-AML had higher impact as it caused peak shifting. AML-BES exerted a more profound effect on disrupting the α-helical structure as it resulted in a higher shift associated with serious decrease in peak intensity and emergence of very clear shoulders at 1645 and 1660 cm^-1^. The former shoulder could be attributed to formation of random coil structure of the protein [21–28] whereas the later could point disorganization of the α-helix. The disorganization of the α-helix results from disruption of some hydrogen bonds in the protein backbone in which amide C = O and N-H groups are involved. This effect is consistent with the fact that the weaker the hydrogen bond involving the amide C = O, the lower the electron density in the C = O group and the higher frequency the amide I absorption appears [22]. The low intensity of the emerged shoulder also emphasizes the formation of weaker hydrogen bonds, as a result of decreased polarization of the amide C = O [6].

A small increase in peak 3 intensity was exhibited in LOS-AML and AML-BES, the latter was also associated with peak shifting to higher frequency. Peaks within this range are usually attributed to β turns [21–28].Accordingly, LOS-AML and AML-BES seems to enhance the formation of this structure.

All of the noticed shifts, shoulders and intensity changes in peaks 1–3 were attributed to be induced by the interaction of the corresponding salt with of the corresponding protein structure since the IR signal of the said salts does not overlap with that of PG SC at these peak positons (Fig 4B–4D).

Peaks 4 and 5 arise from antiparallel β-sheets [21–28]. A small shift to lower frequency was observed in peak 4 as a result of LOS-AML and AML-BES treatments. Both salts exhibited an inherent IR signal at a position to the lower frequency side of peak 4 position (Fig 4C and 4D). It is believed that the noted shift resulted from averaging of the SC and the corresponding salt signals thus not a manifestation of structural changes. Concerning peak 5, it was intensified by AML-BES. Referring to Fig 4D, an intense peak in AML-BES itself arises at the same frequency. This overlap between the endogenous SC and exogenous salt signals makes the effect of the salt on this particular protein form nonconclusive.

Similar to amide I, amide II region showed several changes in peaks positions and intensity. Peaks 1, 2 and 3 within amide II are generally assigned to random coil. Peaks positioned at the higher frequency side of this range can be also assigned to antiparallel β-sheets [29–31]. Referring to Table 1, the increased intensity of peak 1 induced by LOS-K, LOS-AML and AML-BES treatments highlights possible induced formation of random coil and the effect of LOS-K is weaker than that of LOS-AML and AML-BES. The shifts to higher frequency of peak 3 in case of LOS-AML and AML-BES reflects formation of antiparallel β-sheets. On the other hand, peaks 4 and 5 are exhibited in the range that corresponds to the α-helical structure [29–31]. The observed effects of LOS-K, LOS-AML and AML-BES could be brought about by interacting with the protein causing disruption of the α-helix structure. Fig 4B–4D demonstrates that there is no significant interference from the exogenous IR salts of the discussed salts, hence the observed changes can be safely attributed to variations in protein secondary structure.

Depending on the above detailed discussion on the impact of the tested salts on the secondary structure of SC protein component, it can be concluded that the disorganization effect on the α-helical structure and induced formation of parallel β-sheets and random coils were in the order of AML-BES˃LOS-AML˃LOS-K. In addition, the order of the impact of treatments that resulted in increased formation of β-turns was AML-BES˃LOS-AML. The formation of antiparallel β-sheets was manifested by LOS-AML. Thus, the overall effect of these salts on the SC protein was AML-BES˃LOS-AML˃LOS-K. Finally, LOS-DEA had no effect on the protein structure and the α-helical structure of keratin was preserved. These conclusions reflect the trends of inter-sample separation seen in the score plot (Fig 2A). LOS-DEA SC was very similar to the control whereas LOS-K SC was separated from the control but still lies on the same sides of PC 1 and PC 2 as the control. LOS-AML SC was also totally separated and lies on the opposite side of PC1 but same side of PC2 compared to the control. AML-BES SC was also completely separated and did not share any of the PCs sides with any sample. It seems that compared to losartan, amlodipine exerts stronger interaction with the proteins and this interaction is weakened when amlodipine is combined with losartan. Moreover, the extent of losartan interaction with the protein is dependent on its salt type.

In relation to our recent results concerning the retarded percutaneous permeation of losartan and amlodipine from LOS-AML solution compared to LOS-K and AML-BES respectively, the evident interaction of these salts with SC protein could provide the basis for such effect. Possibly, the interaction of losartan and amlodipine with SC when applied individually and not combined helps to modify the barrier properties of SC, thus the interaction is in favor of permeability to some extent. Contrarily, the two drugs when present in combination inside SC, their binding to the protein is enhanced leading to being retained within SC and their permeation hindered. This conclusion is supported by the fact that LOS-DEA provided a permeation that is intermediate between those provided by LOS-K and LOS-AML as shown in Table 2, taking into consideration that the observed differences in Q_24_ values of LOS were statistically significant. By not being able to modify SC protein, LOS-DEA exhibited the base line permeation of LOS, which was enhanced by the interaction of LOS-K with keratin, leading to disorganized structure of the protein and subsequent compromised barrier properties of SC. On the other hand, the more drastic interaction of LOS-AML with keratin could be associated with strong binding of these species with protein component because of their simultaneous presence, consequently hindering their permeation despite their effect on disrupting the protein structural conformation. Pyatski *et al*. (2020) pointed to a similar observation with terbinafine. The interaction of terbinafine with SC keratin, which was evident by induced formation of β-sheet, could regulate its bioavailability in deeper skin layers. The presence of a reservoir of the drug in SC as a result of this interaction was suggested which might allow the controlled released of the drug into the surrounding tissues [16]. On the other hand, the formation of β-sheets induced by hyaluronic acid was suggested to mediate the enhancing effect of hyaluronic acid for permeation of a model protein [17].

**Table 2:** Average Q_24_ in μg of LOS from each corresponding salt.

	Average Q_24_±SD
**LOS-K**	418.58±10.55 [17]
**LOS-DEA**	155.41±46.06
**LOS-AML**	75.07±8.24 [17]

P<0.05

Indeed, our observations hypothesize the potential use of IR microscopy in predicting the permeation of different forms of a drug through SC, thus providing a rapid screening tool prior to conduction of permeation studies saving time, effort and the valuable skin samples.

## 4 Conclusion

The interaction of pharmacologically active drugs with SC biochemical components is underestimated in pharmaceutical research. The nature of this interaction could be crucial for enhancing/retarding the percutaneous permeation of the drug through SC. It could also provide basis for controlled release of the drug through skin. LOS-K was found to enhance the permeation of losartan in a mechanism similar to permeation enhancers that affect the α-helical structure of keratin component of SC. On the other hand, LOS-DEA failed to modify keratin conformation and no signs of interaction with protein were observed by the employed technique. LOS-AML salt impact on the secondary structure of keratin was more drastic than the other two salts of losartan, an effect similar to that of AML-BES. The overall permeation effect of LOS-AML was a retardant for both LOS and AML. Possible binding of these two species to keratin was suggested. Finally, the obtained observation highlight potential use of IR microscopy technique in screening the effect of variable drug forms on the physical barrier properties of SC, and this could be of high value for investigating the percutaneous permeation of substances intended for transdermal/topical delivery.

## Supporting information

S1 File (DOCX)
