# Supplementary material for: IR microspectroscopic investigation of the interaction of some losartan salts with human stratum corneum protein and its effect on losartan transdermal permeation
Source: PLoS One. 2023 Jun 15;18(6):e0287267. doi: 10.1371/journal.pone.0287267 (PMC10270334; doi:10.1371/journal.pone.0287267)
Supplement: S1 File — (DOCX) [file pone.0287267.s001.docx]

**Supplementary Data**

**Characterization of LOS-DEA salt**

**1 Water Content**

The water content of the formed salt, expressed as %w/w from tested sample weight, was done using Karl Fischer titration method (Karl Fischer Titrator, DL38, SRN 5129121684, mix time 15 second, speed 35%, determination weigh > 200 mg, Mettler-Toledo International Inc, Mississauga, Ontario, Canada) in triplicate. The obtained result as the mean ± %RSD was 7.53% **±** 0.35%.

**2 Saturation Solubility**

The saturation solubility was measured for the formed salt in phosphate buffer pH 6.8 and in 60% propylene glycol (PG) in phosphate buffer pH 6.8. The solubility were determined by placing excess salt in 1 ml of the corresponding solvent. The samples were incubated at 37 ± 1℃ at a shaking speed of 200 oscillation/min for 24 hrs followed by centrifugation at 4000 rpm for 15 mins. Aliquots of the resulting supernatants were collected and suitably diluted before injecting onto HPLC to be analyzed for LOS [18]. Each experiment was carried out in triplicate and the results were expressed as the mean ± % RSD. The obtained solubility values in phosphate buffer pH 6.8 and 60% PG in phosphate buffer pH = 6.8 were 220.73 ± 0.27% and 195.56 ± 0.08% respectively.

**3 Apparent Partition Coefficient**

Using each supernatant collected from solubility study in phosphate buffer pH 6.8 and 60% PG in phosphate buffer pH 6.8 (section 2.2), 300 µL were withdrawn and added to 300 µL of 1-octanol followed by incubation at 37± 1℃ and 200 oscillation/min for 24 hrs. After that, aliquots from the aqueous layers were immediately withdrawn and suitably diluted with phosphate buffer pH 6.8. Subsequent analysis for LOS equilibrium concentration in the aqueous samples was done by HPLC [18]. The concentration in the organic phase was calculated from the difference between the initial corresponding concentration of LOS and the equilibrum concentration in the aqueous phase. The results of apparent partition coefficient determination in the form of logP were expressed as the mean of three measurements ± % RSD. The obtained logP values of octanol/phosphate buffer pH 6.8 and octanol/60% PG in phosphate buffer pH = 6.8 were 1.44 ± 0.09% and 0.43 ± 0.10% respectively.

**5 Differential Scanning Calorimetry**

Thermograms for the formed salt in addition to LOS-K was recorded using differential scanning calorimetry (DSC) (NETZSCH DSC 204 F1 Phoenix®, NETZSCH-Gerätebau GmbH, Selb, Bavaria, Germany) over a range of 0 to 300℃ with a heating rate of 10 Kelvin/min. Figure S1 shows the obtained thermograms with evident melting point of the formed salt at 90 ℃.


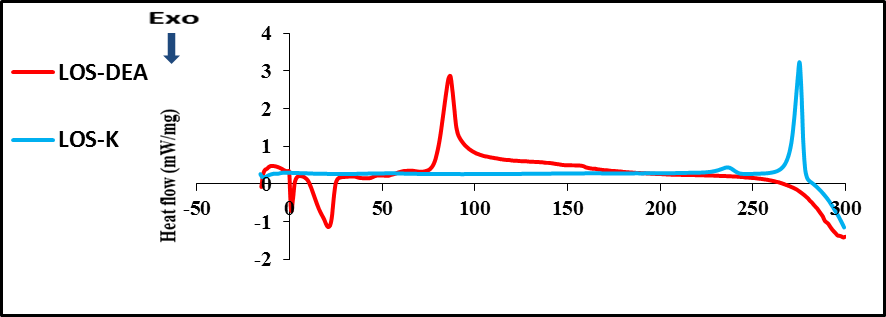


**Temperature (℃)**

**Figure S1.** DSC thermograms of: LOS-K (blue) and LOS-DEA (red).

**5 Proton Nuclear Magnetic Resonance Spectroscopy**

Proton Nuclear Magnetic Resonance (^1^H^_^NMR) spectra were recorded for the formed salt compared to that of parent salt LOS-K using a 500 MHz Bruker NMR spectrometer (Avance III, Bruker, Billerica, Massachusetts, USA). MestreNova-software was employed to construct stacked ^1^H^_^NMR spectra for comparison. Dimethyl sulphoxide (DMSO) and Methanol (MeOD) were used as solvents. The structures of LOS-K and DEA are displayed in figure 2S while figures 3S and 4S show the ^1^H-NMR spectra of the aliphatic and aromatic regions of the studied salts respectively. The frequencies of the distinctive peaks of LOS-K compared to changes appeared in its proposed salt product LOS-DEA are summarized in table 1S.

| 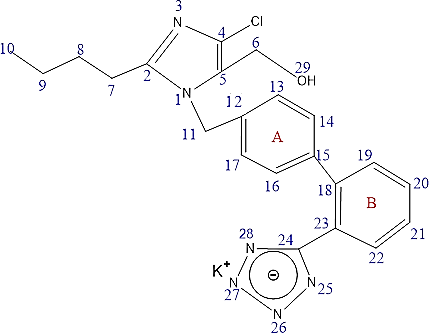 | 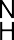 |
| --- | --- |
| **LOS-K** | **DEA** |

**Figure S2.** Chemical structures of LOS-K and DEA.


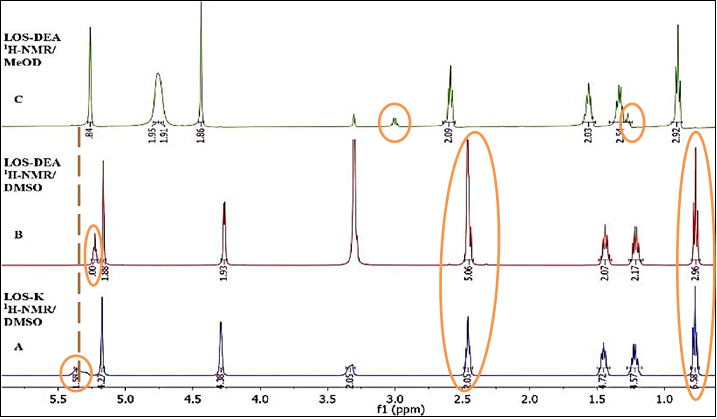
**Figure S3.** ^1^H-NMR spectra of the aliphatic region of LOS-K in DMSO (A), LOS-DEA in DMSO (B) and LOS-DEA in MeOD (C).


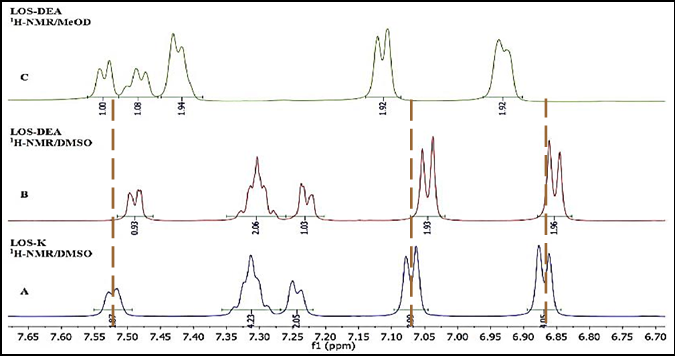


**Figure S4.** ^1^H-NMR spectra of the aromatic region of LOS-K in DMSO (A), LOS-DEA in DMSO (B) and LOS-DEA in MeOD (C).

**Table 1S.** The frequencies of the distinctive peaks of the ^1^H–NMR spectrum of LOS-K compared to changes appeared in its proposed salt product with DEA (LOS-DEA)

| **LOS-K (DMSO)** | **LOS-DEA (DMSO)** |
| --- | --- |
| 0.77 (3H, C10) t | Intensified |
| 1.22 (2H, C9) m | No change |
| 1.45 (2H, C8) m | No change |
| 2.46 (2H, C7) d* | Larger than expected & distorted |
| 3.34 (2H, residual H_2_O) d | Intensified |
| 4.29 (2H, C6) s | No change |
| 5.17 (2H, C11) s | No change |
| 5.36 (1H, OH) s** | - 0.14 & sharpened |
| 6.87 (2H, C13 & C17) d | - 0.02 |
| 7.07 (2H, C14 & C16) d | - 0.02 |
| 7.25 (1H, C19) m | No change |
| 7.32 (2H, C20 & C21) m | No change |
| 7.52 (1H, C22) d | - 0.03 |

(*) peak overlapped with solvent.

(******) broad peak.

DMSO peak (2.50 ppm)

(s) singlet, (d) doublet, (t) triplet and (m) multiplet.

**6 Fourier-Transform Infrared (FTIR) Spectroscopy**

FTIR spectra for LOS and the product (LOS-DEA) were obtained using the KBr disk method. The obtained spectra are displayed in figure 5S whereas a comparison of the corresponding distinctive IR features are summarized in table 2S.


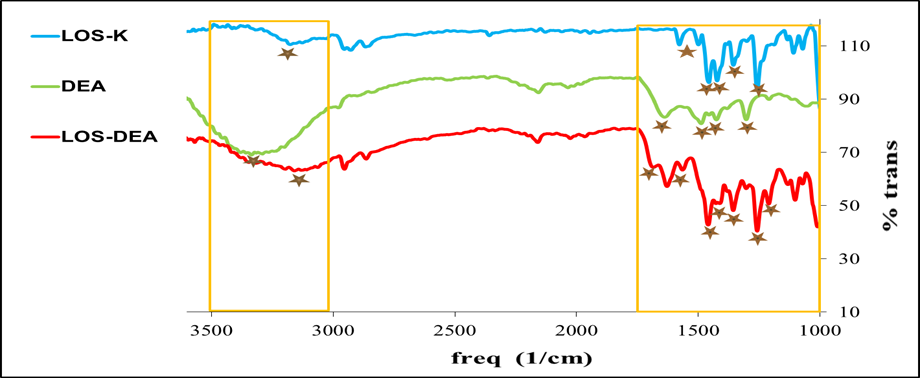


**Figure 5S:** FTIR spectra of: LOS-K (blue), DEA (green) and LOS-DEA (red).
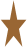
**:** refers to the peaks that showed spectral shifts.

**Table 2S:** The distinctive peaks in the FTIR spectra of LOS-K and DEA, compared to the proposed salt product LOS-DEA.

|  | **Frequency (cm^-1^)** | **Assignment** | **LOS-DEA /Type of change** |
| --- | --- | --- | --- |
| **LOS-K** | ≈ 1250 | Finger print region | - Relatively less intensified |
|  | ≈ 1350 |  | - No change |
|  | ≈ 1400 |  | - Relatively less intensified |
|  | ≈ 1460 |  | - Significantly intensified |
|  | ≈ 1600 & 1500  (jacket) | Aromatic C=C stretching | - Shifted to a higher frequency - Relatively intensified |
|  | very broad band at 3000 - 3300 | O-H (inter-molecular hydrogen bonded perhaps involving H_2_O) stretching | - Shifted to a lower frequency - Significantly broadened (3000 – 3500) - Intensified |
| **DEA** | ≈ 1300 | Finger print region | - Shifted to a lower frequency - Partially overlapped with - the band at about 1250 - Relatively intensified |
|  | ≈ 1410 |  | - Disappeared or overlapped with the band at about 1400 - Relatively intensified |
|  | ≈ 1480 |  | - Disappeared or overlapped with the band at about 1460 - Significantly intensified |
|  | ≈ 1650 | N-H bending | - Shifted to a higher frequency - Partially overlapped with - The band at about 1600 |
|  | very broad band at 3000 -3500 | N-H (secondary amine) stretching | - Disappeared |
